# Bioaccessibility and Antioxidant Capacity of Bioactive Compounds From Various Typologies of Canned Tomatoes

**DOI:** 10.3389/fnut.2022.849163

**Published:** 2022-03-08

**Authors:** Luana Izzo, Luigi Castaldo, Sonia Lombardi, Anna Gaspari, Michela Grosso, Alberto Ritieni

**Affiliations:** ^1^Laboratory of Food Chemistry, Department of Pharmacy, School of Medicine, University of Naples Federico II, Naples, Italy; ^2^Laboratory of Biochemistry and Molecular Biology, Department of Molecular Medicine and Medical Biotechnology, School of Medicine, University of Naples Federico II, Naples, Italy; ^3^CEINGE-Biotecnologie Avanzate, Naples, Italy; ^4^Health Education and Sustainable Development, University of Naples Federico II, Naples, Italy

**Keywords:** canned tomatoes, *in vitro* gastrointestinal digestion, bioaccessibility, antioxidant activity, polyphenol, carotenoid

## Abstract

Tomato (*Solanum lycopersicum* L.) is one of the most consumed vegetables in the world; it contains high amounts of antioxidant phytochemicals and essential nutrients. Although it is commonly consumed fresh, more than 80% of its consumption derives from processed products. Since limited information on changes in the bioaccessibility of bioactive compounds during gastrointestinal digestion was reported, this current study aimed to monitor the antioxidant activity, total polyphenolic and carotenoid content, and bioaccessibility during *in vitro* gastrointestinal digestion of different typologies (*n* = 7) of canned tomatoes. A comprehensive evaluation of the polyphenolic profile of digested and not digested samples was ascertained by ultra-high-performance liquid chromatography combined with high-resolution Orbitrap mass spectrometry. The results highlighted a considerable content of rutin (1.191–9.516 mg/100 g), naringenin (0.359–1.452 mg/100 g), chlorogenic acid (1.857–11.236 mg/100 g), and lycopene (50.894–222.061 mg/kg) in the analyzed matrices. After *in vitro* gastrointestinal digestion, large variability, losses and low recovery were recorded. An appreciable percentage of rutin (30.7%), naringenin (29.6%), chlorogenic acid (25.8%), and lycopene (varied between 9.3 and 20%) remained bioaccessible after the *in vitro* gastrointestinal digestion. Our study could be a valid support to evaluate which content of bioactive compounds could be really bioaccessible to exercise beneficial effects on human health.

## Graphical Abstract

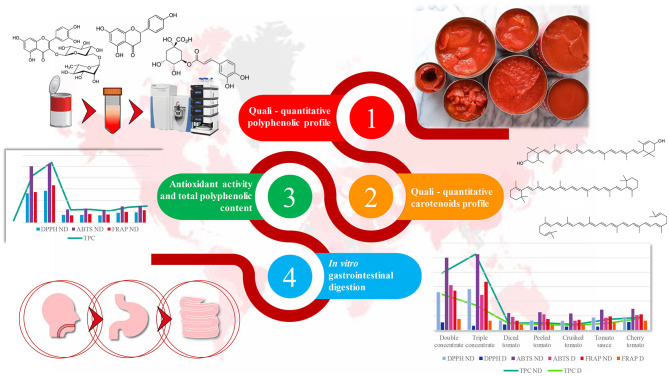

## Introduction

Tomato (*Solanum lycopersicum* L.) is one of the most produced vegetables in the world, with an annual production of 186, 821 million tons. According to latest data reported by the Food and Agriculture Organization (FAO), Italy ranks 4th in world production of tomato and its products, with an annual production of 6,248 million tons, 27.4% of overall European amount in the year 2020. Although it is commonly consumed as fresh, more than 80% of its consumption derives from processed products (1, 2). The Italian consumption of preserved whole tomatoes or in pieces corresponds to 34 g per capita/day (3).

Consumption of fresh or processed tomato plays an important role in nutrition because of well-established health benefits. Tomato is known as a reliable source of biologically active compounds and essential nutrients owing to the array of phenolics and carotenoids it contains (4, 5).

Polyphenols are considered one of the most numerous and widely distributed groups of natural products synthesized by plants (6). There is a broad collection of natural products with similar structural properties that include various subgroups of phenolic compounds, essentially divided into flavonoid and non-flavonoid compounds. Flavonoids contain two benzene rings connected by a 3-carbon linking chain from the nearby pyran ring, whereas phenolic acids, non-flavonoid polyphenolic compounds, are substances composed of a phenolic ring and an organic carboxylic acid function (C6-C1 skeleton) (7, 8). Polyphenols have an important impact on reduction in the risk of chronic degenerative diseases and prevention of cardiovascular heart disease, inflammatory effects, and gastrointestinal disorders (9–11).

Non-nutritive phytochemicals such as lycopene, β-carotene, and lutein belonging to the carotenoid class are also present in significant amounts in tomato (12). Carotenoids constitute a polyene chain that is sometimes terminated by rings and may have additional oxygen atoms attached. Carotenoids are responsible for pigmentation of fruits and vegetables, and play an important role in human health because of their powerful antioxidant potential. In particular, they are associated with anti-inflammation, anti-aging, and anticancer, and they have anti-ulcer capacity as well as other chemoprotective capabilities (13).

As widely reported in literature, tomato contains considerable amounts of phenolic acids and flavonoids, such as rutin, naringenin, chlorogenic acid, and carotenoids. Tomato and its derived products represent major sources of lycopene, which is particularly abundant in ripe tomatoes with a concentration ranging between 30 and more than 200 mg per kg of fresh product (14–16).

To confer beneficial effects on health, bioactive compounds need to be bioaccessible before they are bioavailable to reach target tissues after gastrointestinal (GI) digestion (17). Bioaccessibility is defined as the fraction of nutrients released from the food matrix during GI digestion that is available for absorption (18). Several factors, such as food conservation, cooking, combinations of macronutrients, gastric pH, processing and preservation methods, and lytic enzymes, are able to influence the bioaccessibility of bioactive compounds, which, although they are present in the matrix as such, may not be absorbed (19).

Until now, several analytical approaches are being reported for polyphenol determination in tomato (20–23), such as liquid chromatography coupled to mass spectrometry (LC-MS). Recently, ultra-HPLC combined with high-resolution mass spectrometry has represented an optimal choice for appropriate identification and characterization of compounds with reduction in run time and amelioration in peak shape and accuracy (24–26).

Hence, this current study aimed to investigate the antioxidant activity and total polyphenol content of different typologies (*n* = 7) of commercially canned tomatoes, and evaluate their bioaccessibility during an *in vitro* GI digestion. In addition, qualitative-quantitative profiling of polyphenolic (*n* = 43) and carotenoid (*n* = 3) compounds was performed on extracts and after *in vitro* GI digestion by ultra-high-performance liquid chromatography coupled to high-resolution Orbitrap mass spectrometry and HPLC-diode-array UV/VIS detector analysis, respectively. To the best of the authors' knowledge, this is the first study that investigated these aspects of Italian canned tomatoes.

## Materials and Methods

### Chemicals and Reagents

Water for chromatography (LC-MS grade) (< 18 MΩ/cm resistivity) used for the experiments was acquired from Merck SpA (Milan, Italy). The acetonitrile (Acn), methanol (MeOH), formic acid (FA), acetic acid (AcOH), ethanol (EtOH), hydrochloric acid (HCl), chloroform, and n-hexane of HPLC grade used for analyses were purchased from CARLO ERBA Reagents (Milan, Italy).

All the salts: anhydrous magnesium sulfate (MgSO_4_), sodium chloride (NaCl), potassium thiocyanate (KSCN), sodium bisphosphate (NaH_2_PO_4_) potassium persulfate (K_2_S_2_O_8_), potassium hydroxide (KOH), sodium hydroxide (NaOH), butylhydroxytoluene (BHT), calcium chloride (CaCl_2_), sodium bicarbonate (Na_2_CO_3_), diamine salt of 2,2'-azino-bis (3-ethylbenzothiazolin-6-sulfonic) acid (ABTS), ferrous chloride, 2,4,6-Tris (2-pyridyl)-s-triazine (TPTZ), 2,2-diphenyl-1-picrylhydrazyl (DPPH), Folin-Ciocalteu reagent, Trolox 6-hydroxy−2,5,7,8-tetramethylcroman-2-carboxylic acid; and enzymes: α-amylase (≥ 5 units/mg), pepsin from porcine gastric mucosa (≥ 250 units/mg), pancreatin from porcine pancreas (8 × USP), and bile salts were purchased from Sigma Aldrich (Milan, Italy). Hydrophilic polytetrafluoroethylene (PTFE) syringe filters (15 mm; 0.2 μm) were acquired from Phenomenex (Castel Maggiore, Italy).

### Sampling

Three batches of seven different typologies of canned tomatoes (*n* = 7), which included double and triple tomato concentrates, diced tomatoes, peeled tomatoes, crushed tomatoes, tomato sauce, and cherry tomatoes were analyzed. Double and triple concentrate, sauce, crushed are produced from Parma round tomato variety. Peeled tomatoes are produced from the long tomato variety. Diced tomatoes are produced from the “Datterino” tomato variety. Cherry tomatoes are produced from cherry tomato variety. All the typologies were produced with 100% Italian tomatoes and were acquired from various shops located in Campania region, Italy. After arrival in the laboratory, all the samples were properly stored at room temperature in original packaging, and the analyses were carried out before the expiration date. Prior to the analyses, the canned tomato samples were homogenized with an Ultra Turrax® instrument (T 25 digital ULTRA-TURRAX®) to obtain a homogeneous sample from all parts.

### Moisture Content

Determination of moisture content of the canned tomato samples was performed according to the protocol reported in (4). In short, 5 g of the samples was dried at 70 °C using a laboratory oven for 6 h. Moisture content was determined by weighing the samples after drying. Data were expressed as g/100 g of samples.

### Extraction of Polyphenol Compounds From Canned Tomatoes

Extraction of polyphenols was carried out following the procedure reported in (23), with some modifications. Following this method, 5 mL of 80% ethanol acidified with 0.1% formic acid was added to 2 g of the samples. The mixture was mixed for 1 min and subsequently sonicated for 5 min. A freezer pack was placed in a water bath to prevent degradation of bioactive compounds. Afterward, the samples were centrifuged (X3R Heraeus Multifuge, Thermo Fisher Scientific) for 5 min, at 2,800 × g at 4°C. The supernatant was recovered and kept, and the pellet was re-extracted. The pooled supernatant (about 10 mL) was dried using a nitrogen evaporator (Laborata 4000; Heidolph Instruments Italia Srl, Milan, Italy) and then reconstituted with 2 mL of water acidified to 0.1% formic acid. Finally, the extract was filtered with 0.22 μm nylon filters and was ready for successive analyses.

### UHPLC Q-Orbitrap HRMS

Polyphenol determination was performed as previously described in (27). The analysis was performed using a UHPLC system (Dionex UltiMate 3000; Thermo Fisher Scientific, Waltham, MA, United States) equipped with a degassing system, a quaternary UHPLC pump, and an autosampler device. Chromatography separation was accomplished with a thermostated (25°C) Kinetex F5 (50 mm × 2.1 mm, 1.7 μm) column (Phenomenex, Castel Maggiore, Italy). Mobile phases consisted of H_2_O containing 0.1% formic acid (A) and MeOH containing 0.1% formic acid (FA) (B). Chromatographic separation was carried out under the following conditions: 0–0.5 min, 0% B; 0.5–1 min, 0–40% B; 1–2 min, 40–80% B; 2–5 min, 80–100% B; 5–9 min, 100% B; 9–11 min, 100–0% B; 11–13 min, 0% for column re-equilibration. Flow rate was set at 500 μL/min and injection volume at 5 μL. Detection was performed using a Q-Exactive Orbitrap mass spectrometer (Thermo Fisher Scientific, Waltham, MA, United States) operated in positive and negative modes. Full ion MS and all ion fragmentation (AIF) scan events were set. The following settings were fixed in full MS scan mode: scan range 80–1,000 *m/z*, resolution power of 70,000 full width at half maximum, microscan 1, automatic gain control target 1 × 10^6^, maximum injection time 200 ms, sheath gas flow rate 18, auxiliary gas 3, sweep gas flow rate 0, spray voltage 3.5 KV, capillary temperature 320°C; S-lens RF level 60, and auxiliary gas heater temperature 350°C. In the AIF scan mode, the resolution power was set as: scan range 80–1,000 *m/z*, resolution power of 17,500 full width at half maximum, microscan 1, automatic gain control target 1 × 10^5^, maximum injection time 200 ms, sheath gas flow rate 18, auxiliary gas 3, sweep gas flow rate 0, spray voltage 3.5 kV, capillary temperature 320°C, S-lens RF level 60, and auxiliary gas heater temperature 350°C. Collision energy (CE) was set at 15, 30, and 45 eV to achieve a representative product ion spectrum. Mass tolerance was fixed at 5 ppm for identification and confirmation of target molecular ions and at 1 ppm for retrospective analysis of data; scan time = 0.10 s and retention time to 30 s. Data processing was performed using the Quan/Qual Browser Xcalibur software 3.1.66.19 (Xcalibur; Thermo Fisher Scientific, Waltham, MA, United States).

### Carotenoid Extraction

Extraction of carotenoids was performed following the protocol in (28), with slight modifications. The procedure involves extraction of 1 g of samples, with 6 mL of 0.1% BHT ethanol. After 1 min of vortex, the samples were incubated in a water bath for 5 min at 85°C. Then, 120 μL of an 80% aqueous KOH solution was added, vortexed for 1 min, and re-incubated for saponification for 10 min. Then, the samples were cooled for 5 min in a freezer at −80°C. Addition of 3 mL of hexane and 3 mL of water is followed by centrifugation (X3R Heraeus Multifuge; Thermo Fisher Scientific) for 5 min at 2,800 × g. After centrifugation, the hexane phase was collected. The extraction procedure was repeated two more times, and the supernatants were combined, dried with nitrogen, resuspended in 1 mL of chloroform, and filtered with 0.2-μm nylon filters before analysis.

### HPLC-Diode-Array UV/Vis Detector (DAD) Analysis

Carotenoid analysis was performed on the Jasco HPLC Model 2000 Plus Series (Jasco, Cremella, Italy) equipped with a pump (PU-2080), a UV-Vis detector (UV-2075 Plus; Jasco), and an autosampler (AS-2055 Plus; Jasco,). Chromatography separation was carried out using a Gemini C18 (250 mm × 4.6 mm, 100 A, 5 μm) column (Phenomenex, Castel Maggiore, Italy). Mobile phases consisted of acetonitrile as solvent system A and *n-*hexane, ethanol, and dichloromethane (1:1:1) as mobile phase B. Chromatographic separation was carried out under the following conditions: initial 18% B, increased to 24% B in 8 min, to 42% B in 4 min, and to 61% B in 6 min. The gradient was reduced to 18% B in 4 min and another 5 min for column re-equilibration at 18%. Total run time was 27 min, and flow rate was 1 mL/min. The absorbance of lutein, β-carotene, and lycopene was measured at 450 nm.

### *In vitro* GI Digestion

*In vitro* gastrointestinal digestion was performed following the protocol reported in (29), with slight modifications. The *in vitro* GI digestion includes three steps: the oral, gastric, and intestinal phases. To simulate the oral phase, 5 g of the samples were combined with 3.5 mL of simulated salivary fluid (SSF), 500 μL of a-amylase solution (1,500 U/mL in SSF), 25 μL of 0.3 M calcium chloride dihydrate, and 0.975 mL of distilled water. Then, the pH was adjusted to 7 using HCl 1 M, and the mixture was incubated at 37°C for 2 min at 150 rpm in a water bath orbital shaker (GFL-1086; Biosigma S.p.A., Venice, Italy).

The oral stage continues with the gastric phase, in which 7.5 mL of simulated gastric fluid (SGF), 1.6 mL of a pepsin solution (25,000 U/mL in SGF), 5 μL of 0.3 M calcium chloride dihydrate, and 0.695 mL of distilled water were added. In this step, the pH was adjusted to 3 with HCl 6 M, and the mixture was incubated at 37°C for 2 h at 150 rpm in the water bath orbital shaker (GFL-1086; Biosigma S.p.A., Venice, Italy).

Finally, in the intestinal phase, 11 mL of simulated intestinal fluid (SIF), 5 mL of pancreatin solution (800 U/mL), 2.5 mL of bile salts (160 mM), 40 μL of 0.3 M calcium chloride dihydrate, and 1,310 μL of distilled water were added. It is suggested to verify the pH value and adjust it to 7 using NaOH 6 M. The mixture was incubated at 37°C for 2 h at 150 rpm in the water bath orbital shaker (GFL-1086; Biosigma S.p.A., Venice, Italy).

At the end of the intestinal phase, the mixture was centrifuged using X3R Heraeus Multifuge; (Thermo Fisher Scientific) for 5 min at 2,800 × g. The supernatants were collected and freeze-dried, resuspended in methanol, and centrifuged for min at 5,000 rpm; the supernatants without salts were used for further experiments.

The preparation of SSF, SGF, and SIF is schematized in a Table of our previously published scientific study (30).

### Total Phenolic Content

Total phenolic content (TPC) was measured on the diluted extract following the method reported by (31). In short, 0.125 mL of polyphenolic extract was added to 0.5 mL of deonized water and 0.125 mL of the Folin–Ciocalteu reagent. After 6 min of incubation, 1.25 mL of 7.5% sodium carbonate solution and 1 mL of deionized water were added to the mixture. After 90 min of incubation under dark conditions and at room temperature, the absorbance was recorded at 760 nm. Autozero was carried out with distilled water. All the experiments were conducted in triplicate. The results were expressed in mg gallic acid equivalent (GAE)/100 g of fresh weight.

### Antioxidant Activity

Antioxidant activity was measured by three different assays: the DPPH, ABTS, and FRAP tests. All the experiments were conducted in triplicate. The results were expressed in mmol Trolox/kg of fresh weight.

### DPPH Assay

Radical-scavenging activity was determined using the method suggested by (56), with slight modifications. Briefly, 2 mg of DPPH salt were diluted with methanol until to reach an absorbance value at 515 nm of 0.9 (±0.02). Once the working solution was obtained, to 1 mL of the DPPH∙+ solution, we added 200 μL of the diluted sample. Absorbance was measured after waiting for 10 min. Autozero was carried out with methanol.

### ABTS Assay

Free radical-scavenging activity was determined with the method previously reported by (32). In short, 9.6 mg of ABTS salt was dissolved in 2.5 mL of deionized water. To this mixture, we added 44 μL of potassium persulfate. The solution was maintained under dark conditions at 4°C for 16 h prior to use. Then, the solution was diluted in ethanol until an absorbance value at 734 nm of 0.7 (±0.02). Once the working solution was obtained, to 1 mL of the ABTS^∙+^ solution, we added 100 μL of the diluted sample. The absorbance was rapidly measured after waiting for 2.5 min. Autozero was carried out with ethanol.

### FRAP Assay

Ferric reducing antioxidant power (FRAP) was measured based on the protocol reported in (33), with slight changes. A FRAP solution was prepared by mixing a TPTZ solution (10 mM, in HCl 40 mM), a ferric chloride solution (20 mM, in water), and an acetate buffer (0.3 M; pH 3.6) with a ratio of 1:1:10 (*v/v/v*). In short, 150 μL of diluted samples were added to 2,850 mL of the FRAP solution. The absorbance was recorded after 4 min at 593 nm. Autozero was carried out with methanol.

### Statistical Analysis

The results were expressed as average ± standard deviation (SD) evaluated on three independent replication. Tukey's test was performed to evaluate differences among the different typologies of tested samples. Tukey's test at a level of *p* < 0.05 was considered significant. Statistical analysis was performed using the software Info-Stat version 2008 (https://www.infostat.com.ar/index.php?mod=page&id=15).

## Results

### Moisture Content

The moisture content obtained by gravimetric analysis is summarized in Table 1. The moisture content found in different typologies of the tomato samples ranged from 63.8 to 90.4 g/100 g of sample.

**Table 1:** Levels of moisture content found in the assayed canned tomato samples.

**Sample**	**Moisture content**	
	**g/100 g**	**±SD**
Double concentrate	74.7^a^	0.5
Triple concentrate	63.8^b^	0.4
Diced tomato	89.4^c^	0.5
Peeled tomato	90.4^c^	0.7
Crushed tomato	89.8^c^	0.8
Tomato sauce	88.8^c^	0.8
Cherry tomato	89.6^c^	0.7

a−c *Different letters show significant difference (p < 0.05) among the different typologies of canned tomatoes*.

### Quantification and Retrospective Analysis of Polyphenol Compounds in Canned Tomatoes by UHPLC-Q-Exactive HRMS

Bioactive compounds of canned tomato extracts were profiled by UHPLC-Q-Orbitrap HRMS. A total of 25 different polyphenolic compounds such as flavonoids and phenolic acids were investigated by combining MS and MS/MS spectra (Table 2). Analysis of phenolic acids and flavonoids was performed in ESI, producing the deprotonated molecular ion [M-H]. Identification was carried out by comparison to their relative reference pure standards. Quantitative –determination was performed through calibration curves at nine concentration levels (5–0.019 μg/kg).

**Table 2:** Chromatographic and spectrometric parameters: retention time (RT), chemical formula, theoretical and measured masses (*m/z*), accuracy, and sensibility for phenolic acids and flavonoids (*n* = 25) in the investigated canned tomato samples.

**Analyte**	**RT (min)**	**Chemical formula**	**[M-H]^**−**^theoretical mass (*m/z*)**	**[M-H]^**−**^found mass (*m/z*)**	**MS/MS fragment ions (*m/z*)**	**Accuracy (Δ ppm)**	**LOD (mg/kg)**	**LOQ (mg/kg)**
Protocatechuic acid	2.41	C_7_H_6_O_4_	153.01930	153.01857	109.02840	−4.77064	0.026	0.078
Epicatechin	2.98	C_15_H_14_O_7_	289.07176	289.07202	221.94647–203.09201–161.04478	0.89943	0.013	0.039
Caffeic acid	3.05	C_9_H_8_O_4_	179.03498	179.03455	134.99960	−2.40177	0.013	0.039
Vanillic acid	3.07	C_8_H_8_O_4_	167.03490	167.03428	151.03905–123.04387	−3.71180	0.026	0.078
Chlorogenic acid	3.11	C_16_H_18_O_9_	353.08780	353.08798	191.05594–84.98998	0.50979	0.013	0.039
Catechin	3.18	C_15_H_14_O_6_	289.07175	289.07205	247.02241–205.10712–151.03923–125.02335	1.03780	0.026	0.078
Daidzein	3.29	C_15_H_9_O_4_	253.05063	253.04977	209.96429–225.00984	−3.39853	0.052	0.156
*p-*coumaric acid	3.31	C_9_H_8_O_3_	163.04001	163.03937	119.04917	−3.92542	0.026	0.078
Ferulic acid	3.38	C_10_H_10_O_4_	193.05063	193.05016	178.02666–149.06009-−134.99963	−2.43459	0.013	0.039
Syringic acid	3.39	C_9_H_10_O_5_	197.04555	197.04503	182.02153–166.99791	−2.63898	0.026	0.078
Genistin	3.40	C_15_H_10_O_5_	269.04554	269.04562	241.14435–213.14908–151.03935	0.29735	0.013	0.039
Isoquercetin	3.51	C_21_H_20_O_12_	463.08820	463.08853	431.09848–187.09698–174.95542	0.71261	0.013	0.039
Rutin	3.55	C_27_H_30_O_16_	609.14611	609.14673	300.99911–271.05026–255.12390	1.01782	0.013	0.039
Naringin	3.56	C_27_H_32_O_14_	579.17193	579.17212	515.11951–477.10406–463.08841–359.07724	0.32805	0.026	0.078
Quercetin 3-glucoside	3.59	C_21_H_20_O_12_	463.08820	463.08817	447.09344–359.07730	−0.06478	0.026	0.078
Vitexin	3.58	C_21_H_20_O_10_	431.09837	431.09824	317.03000–174.95531	−0.30156	0.026	0.078
Diosmin	3.60	C_28_H_32_O_15_	607.16684	607.16711	593.15240–463.08835–447.09323–317.03027	0.44469	0.013	0.039
Ellagic acid	3.61	C_14_H_6_O_8_	300.99899	300.99911	245.91669-229.93712-185.01208-117.00336	0.39867	0.013	0.039
Isorhamnetin 3-rutinoside	3.62	C_28_H_32_O_16_	623.16117	623.16223	507.10849–447.09338–317.03012–253.05043	1.70100	0.026	0.078
Kaempferol 3-glucoside	3.63	C_21_H_20_O_11_	447.09328	447.09332	300.99915-273.07690-227.07104	0.08947	0.026	0.078
Myricetin	3.64	C_15_H_10_O_8_	317.03029	317.03040	300.99899–253.05046–128.95857	0.34697	0.013	0.039
Quercetin	3.75	C_15_H_10_O_7_	301.03538	301.03508	174.95551	−0.99656	0.013	0.039
Naringenin	3.80	C_15_H_12_O_5_	271.06120	271.06110	235.92595–151.03917	−0.36892	0.013	0.039
Kaempferol	3.86	C_15_H_10_O_6_	285.04046	285.04086	93.00679	1.40331	0.013	0.039
Apigenin	3.93	C_15_H_10_O_5_	269.04555	269.04550	248.96060–174.95537–91.00249	−0.18584	0.013	0.039

Results of the quantitative analysis are shown in Table 3. Predominant compounds found in all the studied typologies of canned tomatoes were represented by rutin, naringenin, and chlorogenic acid. In particular, chlorogenic acid was in the range of 1.857–11.236 mg/100 g, rutin ranged between 1.191 and 9.516 mg/100 g, and naringenin ranged from 0.359 to 1.452 mg/100 g. As far as phenolic acids are concerned, chlorogenic acid was the compound quantified with highest concentration in double- and triple-concentrated canned tomatoes, followed by the cherry tomato typology, with an average value of 4.852 mg/100 g. With regard to flavonoids, rutin and naringenin were found to have an average value of 4.215 and 0.808 mg/100 g, respectively. With regard to concentration of the other investigated polyphenols, great variability was recorded among the typologies, and the amount determined was significantly lower (*p* < 0.05).

**Table 3:** Quantitative analysis of bioactive compounds in the investigated canned tomato extracts (*n* = 7) performed by UHPLC-Q-Orbitrap HRMS analysis.

**Analyte**	**Double concentrate**		**Triple concentrate**		**Diced tomato**		**Peeled tomato**		**Crushed tomato**		**Tomato sauce**		**Cherry tomato**	
	**Mean**	**±SD**	**Mean**	**±SD**	**Mean**	**±SD**	**Mean**	**±SD**	**Mean**	**±SD**	**Mean**	**±SD**	**Mean**	**±SD**
Protocatechuic acid	0.001^a^	0.000	0.002^b^	0.000	0.002^b^	0.000	0.002^b^	0.000	0.002^b^	0.000	0.003^c^	0.000	0.001^a^	0.000
Chlorogenic acid	6.597^a^	0.967	7.112^a^	0.756	3.730^b^	0.143	1.857^c^	0.283	3.432^d^	0.119	5.048^e^	0.494	11.236^f^	0.567
Caffeic acid	0.745^a^	0.050	0.894^b^	0.016	0.189^c^	0.003	0.205^c, d^	0.023	0.204^c, d^	0.002	0.292^d, e^	0.097	0.355^e^	0.011
*p*-coumaric acid	0.060^a^	0.001	0.080^b^	0.002	0.045^c^	0.001	0.036^d^	0.001	0.048^c^	0.002	0.056^e^	0.002	0.060^a, e^	0.002
Ferulic acid	0.450^a^	0.080	0.470^a^	0.089	0.408^a^	0.065	0.256^b^	0.050	0.350^a^	0.041	0.411^a^	0.036	0.430^a^	0.048
Genistin	0.015^a^	0.003	0.015^a^	0.002	0.080^b^	0.003	0.050^c^	0.003	0.080^b^	0.002	0.080^b^	0.001	0.080^b^	0.000
Naringin	0.080^a^	0.001	0.100^b^	0.003	0.096^b^	0.001	0.092^c^	0.001	0.087^d^	0.001	0.090^c^	0.001	0.080^a^	0.001
Quercetin 3-glucoside	0.002^a^	0.000	0.002^a^	0.000	0.000^b^	0.000	0.001^c^	0.000	0.001^c^	0.000	0.001^c^	0.000	0.001^c^	0.000
Kaempferol 3-glucoside	0.003^a^	0.000	0.004^b^	0.000	0.001^c^	0.000	0.001^c^	0.000	0.001^c^	0.000	0.001^c^	0.000	0.002^d^	0.000
Rutin	7.905^a^	0.321	9.516^b^	0.167	2.248^c^	0.058	1.191^d^	0.003	1.441^e^	0.051	3.481^f^	0.202	3.722^f^	0.059
Vitexin	0.123^a^	0.015	0.156^a^	0.018	0.050^b^	0.001	0.056^c^	0.002	0.059^c^	0.001	0.065^d^	0.001	0.089^e^	0.013
Isorhamnetin 3-rutinoside	0.037^a^	0.002	0.045^b^	0.001	0.009^c^	0.000	0.010^c^	0.001	0.010^c, d^	0.000	0.015^d, e^	0.005	0.018^e^	0.001
Myricetin	0.016^a^	0.001	0.019^b^	0.001	* < loq*	-	* < loq*	-	* < loq*	-	* < loq*	-	0.016^a^	0.000
Naringenin	1.202^a, d^	0.123	1.452^a^	0.163	0.426^b^	0.002	0.359^c^	0.050	0.530^c^	0.140	0.590^c^	0.097	1.103^d^	0.056
Kaempferol	0.005^a, b^	0.001	0.007^a^	0.001	* < loq*	-	* < loq*	-	* < loq*	-	* < loq*	-	0.003^b^	0.001
Quercetin	0.013^a^	0.001	0.018^b^	0.002	0.010^c^	0.000	0.010^c^	0.000	0.009^c^	0.000	0.009^c^	0.000	0.013^a^	0.001
Apigenin	0.010^a^	0.000	0.013^b^	0.001	0.009^a^	0.000	0.006^c^	0.000	0.006^c^	0.000	0.008^d^	0.000	0.009^a^	0.000

*Results are expressed in mg/100 g of fresh weight and reported as mean ± SD from three independent experiments*.

a−f *Different letters show a significant difference (p < 0.05) among the different typologies of canned tomatoes*.

Retrospective analysis allowed for the identification and semi-quantification of 18 further polyphenolic compounds (Table 4). For the quantitative analysis of compounds, for which a reference standard was not available, a representative standard of the same group was selected (rutin and quercetin 3-glucoside). The most representative compounds were represented by caffeic acid hexoside (range 0.101–0.68 mg/100 g; average 0.384 mg/100 g), cryptochlorogenic acid (range 0.094–1.076 mg/100 g; average 0.566 mg/100 g), caffeic acid diesoside (range 0.101–0.68 mg/100 g; average 0.384 mg/100 g), rutin O-pentoside (range 0.106–1.271 mg/100 g; average 0.506 mg/100 g), and floretin diglycoside (range 0.067–0.755 mg/100 g; average 0.426 mg/100 g).

**Table 4:** Retrospective data analysis, identification, and semi-quantitative analysis of 18 no-target polyphenols in the different types of analyzed canned tomato samples (*n* = 7).

**Analyte**	**Double concentrate**		**Triple concentrate**		**Diced tomato**		**Peeled tomato**		**Crushed tomato**		**Tomato sauce**		**Cherry tomato**	
	**Mean**	**±SD**	**Mean**	**±SD**	**Mean**	**±SD**	**Mean**	**±SD**	**Mean**	**±SD**	**Mean**	**±SD**	**Mean**	**±SD**
Protocatechuic acid O-hexoside	0.032^a^	0.001	0.042^b^	0.002	0.020^c, d^	0.002	0.017^c^	0.001	0.023^d^	0.001	0.021^d^	0.001	0.017^c^	0.001
Vanillic acid hexoside	0.063^a^	0.000	0.067^a^	0.010	0.021^b^	0.000	0.014^c^	0.000	0.018^d^	0.000	0.026^e^	0.000	0.047^f^	0.000
Coumaric acid hexoside	0.304^a^	0.015	0.332^b^	0.005	0.040^c^	0.002	0.018^d^	0.000	0.079^e^	0.001	0.422^f^	0.131	0.526^f^	0.030
Caffeic acid hexoside	0.580^a^	0.009	0.548^b^	0.005	0.206^c^	0.004	0.101^d^	0.006	0.178^e^	0.009	0.326^f^	0.077	0.680^g^	0.008
Cryptochlorogenic acid	0.695^a, f^	0.151	1.076^b^	0.047	0.554^a^	0.008	0.094^c^	0.003	0.192^d^	0.010	0.528^e^	0.013	0.822^f^	0.090
Rutinhexoside	0.027^a^	0.002	0.019^b^	0.000	0.009^c^	0.000	0.003^d^	0.001	0.010^e^	0.000	0.051^f^	0.001	0.026^a^	0.005
Cumaroylquinic acid	0.072^a^	0.000	0.025^b^	0.000	0.042^c^	0.000	0.021^d^	0.001	0.042^c^	0.000	0.041^c^	0.002	0.034^e^	0.001
Feruloylquinic acid	0.008^a^	0.000	0.012^b^	0.000	0.006^c^	0.000	0.008^a^	0.000	0.006^c^	0.000	0.006^c^	0.000	0.008^a^	0.000
Naringenin C hexoside	0.436^a^	0.005	0.562^b^	0.005	0.104^c^	0.006	0.120^d^	0.005	0.094^c^	0.004	0.201^e^	0.005	0.200^e^	0.004
Eriodicthiol	0.180^a^	0.005	0.210^b^	0.006	0.083^c^	0.002	0.053^d^	0.003	0.039^e^	0.004	0.048^f^	0.004	0.095^g^	0.004
Tricaffeoilquinic acid	0.031^a^	0.001	0.035^b^	0.002	0.025^c^	0.004	0.023^c^	0.003	0.024^c^	0.002	0.026^c^	0.001	0.027^c^	0.003
Caffeic Acid dihexoside^*^	0.580^a^	0.009	0.614^b^	0.005	0.206^c^	0.004	0.101 ^d^	0.006	0.178^e^	0.009	0.326^f^	0.077	0.680^g^	0.008
Naringenin C diglycoside^*^	0.432^a^	0.015	0.583^b^	0.016	0.160^c^	0.021	0.046^d^	0.002	0.061^e^	0.008	0.105^f^	0.002	0.137^c^	0.007
Eriodicthiol O hexoside ^*^	0.109^a^	0.002	0.156^b^	0.000	0.019^c^	0.000	0.022^d^	0.001	0.013^e^	0.000	0.019^c^	0.001	0.035^f^	0.003
Quercetin O dihexoside ^*^	0.034^a^	0.001	0.046^b^	0.001	0.009^c^	0.000	0.039^d^	0.001	0.015^e^	0.001	0.017^e^	0.001	0.026^f^	0.000
Rutin O pentoside ^*^	1.009^a^	0.006	1.271^b^	0.047	0.147^c^	0.011	0.106^d^	0.003	0.201^e^	0.004	0.398^f^	0.035	0.411^g^	0.021
Floretindiglycoside ^*^	0.662^a^	0.031	0.755^b^	0.030	0.233^c^	0.041	0.067^d^	0.004	0.306^f^	0.036	0.363^f^	0.080	0.596^g^	0.025
Kaempferol 3-O-rutinoside ^*^	0.079^a^	0.004	0.155^b^	0.001	0.060^c^	0.002	0.025^d^	0.000	0.027^e^	0.000	0.077^a^	0.006	0.094^f^	0.003

*Results are expressed in mg/100 g of fresh weight and reported as mean ± SD from three independent experiments*.

a−g *Different letters show a significant difference (p < 0.05) among the different typologies of canned tomatoes*.

* *Semi-quantification with rutin*.

### Bioaccessibility of Polyphenol Compounds in Canned Tomatoes by UHPLC-Q-Exactive HRMS

Polyphenolic profile was followed during *in vitro* GI digestion by UHPLC Q-Orbitrap HRMS. The results are reported in Table 5. In particular, the analysis was conducted in the intestinal stage. Among the various analyzed compounds, there were large variability and losses, and low recovery amount was obtained (7.9–69.7%). Rutin was recovered at a percentage of 30.7% (range: 21–45.1%), naringenin at a parcentage of 29.6% (range 18.9–38%), and chlorogenic acid at a percentage of 25.8% (range 15.1–32.9%). Quercetin 3-glucoside and Kaempferol 3-glucoside were not bioaccessible during the GI tract digestion. For protocatechuic acids, Kaempferol and myricetin were reported to have low recovery in the analyzed intestinal stage.

**Table 5:** Quantitative analysis of bioactive compounds in the investigated canned tomato samples (*n* = 7) in the intestinal stage performed by UHPLC-Q-Orbitrap HRMS analysis.

**Analyte**	**Double concentrate**		**Triple concentrate**		**Diced tomato**		**Peeled tomato**		**Crushed tomato**		**Tomato sauce**		**Cherry tomato**	
	**Mean**	**±SD**	**Mean**	**±SD**	**Mean**	**±SD**	**Mean**	**±SD**	**Mean**	**±SD**	**Mean**	**±SD**	**Mean**	**±SD**
	**Intestinal phase**													
Protocatechuic acid	0.000^a^	0.000	0.001^b^	0.000	-	-	-	-	0.001^b^	0.000	0.001^b^	0.000	0.000^a^	0.000
Chlorogenic acid	1.100^a^	0.120	2.341^b^	0.126	0.963^c^	0.024	0.280^d^	0.047	1.123^a^	0.020	1.452^e^	0.082	3.256^f^	0.210
Caffeic acid	0.169^a^	0.104	0.287^a^	0.131	0.051^b, d^	0.001	0.045^b, d^	0.005	0.058^c, d^	0.000	0.063^d, e^	0.022	0.120^e^	0.056
*p*-coumaric acid	0.008^a^	0.000	0.026^b^	0.006	0.011^c^	0.000	0.007^d^	0.000	0.009^e^	0.000	0.018^f^	0.000	0.027^b, f^	0.011
Ferulic acid	0.059^a^	0.018	0.145^b^	0.020	0.097^c^	0.014	0.049^a^	0.014	0.114^c^	0.009	0.142^b^	0.008	0.186^d^	0.012
Genistin	0.003^a^	0.001	0.005^b^	0.000	0.018^c^	0.001	0.001^a^	0.001	0.025^d^	0.000	0.028^d^	0.003	0.038^e^	0.008
Naringin	0.021 ^a^	0.009	0.032^b^	0.001	0.023^a^	0.000	0.023^a^	0.000	0.024^a^	0.000	0.034^b^	0.004	0.032^b^	0.009
Quercetin 3-glucoside	-	-	-	-	-	-	-	-	-	-	-	-	-	-
Kaempferol 3-glucoside	0.001^a^	0.000	0.001^a^	0.000	-	-	-	-	-	-	-	-	-	-
Rutin	1.658^a^	0.071	3.154^b^	0.037	0.543^c^	0.013	0.256^d^	0.013	0.426^e^	0.011	1.568^a^	0.045	1.520^a^	0.130
Vitexin	0.024^a^	0.002	0.048^b^	0.004	0.013^c^	0.000	0.001^d^	0.001	0.017^e^	0.000	0.018^e^	0.002	0.041^b^	0.009
Isorhamnetin 3-rutinoside	0.008^a^	0.001	0.008^a^	0.000	0.002^b^	0.000	0.002^b^	0.000	0.002^b^	0.000	0.001^b^	0.001	0.008^a^	0.002
Myricetin	0.003^a^	0.000	0.006^b^	0.000	*-*	-	*-*	-	*-*	-	*-*	-	0.004^c^	0.000
Naringenin	0.404^a^	0.024	0.478^b^	0.032	0.098^c^	0.000	0.068^d^	0.000	0.168^e^	0.000	0.224^f^	0.023	0.321^a, f^	0.140
Kaempferol	0.001^a^	0.000	0.002^a^	0.000	*-*	-	*-*	-	*-*	-	*-*	-	0.001^a^	0.000
Quercetin	0.002^a^	0.000	0.005^b^	0.000	0.003^c^	0.000	0.002^a^	0.011	0.002^a^	0.000	0.003^a, c^	0.001	0.004^c^	0.000
Apigenin	0.002^a^	0.000	0.004^b^	0.000	0.002^a^	0.000	0.001^a^	0.000	0.002^a^	0.000	0.002^a^	0.000	0.003^b^	0.000

*The results are expressed in mg/100 g of fresh weight and reported as mean ± SD from three independent experiments*.

a−f *Different letters show a significant difference (p < 0.05) among the different typologies of canned tomatoes*.

Assessment of bioaccessibility was also performed during GI digestion for semi-quantified compounds. The results reported in Table 6 show that coumaric acid hexoside, caffeic acid hexoside, cryptochlorogenic acid, naringenin C hexoside, rutin O-pentoside, and floretindiglycoside were the most abundant compounds. After GI digestion, rutin hexoside was recovered at a percentage of 53.6%, rutin O-pentosideat a percentage of 63.3% and caffeic acid hexoside at a percentage of 42.2%. Among the other investigated typologies, cherry tomatoes showed comparable concentrations to those of double and triple concentrates for most of the studied analytes.

**Table 6:** Retrospective data analysis, identification, and semi-quantitative analysis of 18 no-target polyphenols in the different types of analyzed samples (*n* = 7) in the intestinal stage performed by UHPLC-Q-Orbitrap HRMS analysis.

**Analyte**	**Double concentrate**		**Triple concentrate**		**Diced tomato**		**Peeled tomato**		**Crushed tomatoes**		**Tomato sauce**		**Cherry tomato**	
	**Mean**	**±SD**	**Mean**	**±SD**	**Mean**	**±SD**	**Mean**	**±SD**	**Mean**	**±SD**	**Mean**	**±SD**	**Mean**	**±SD**
	**Intestinal phase**													
*Protocatechuic acid O-hexoside*	0.015^a^	0.001	0.025^b^	0.001	0.006^c^	0.000	0.007^c^	0.000	0.008^d^	0.000	0.008^d^	0.001	0.013^a^	0.001
*Vanillic acid hexoside*	0.015^a^	0.001	0.017^b^	0.000	0.002^c^	0.000	0.016^b^	0.001	0.008^d, e^	0.001	0.007^d^	0.000	0.009^e^	0.000
*Coumaric acid hexoside*	0.160^a^	0.001	0.202^b^	0.008	0.025^c^	0.001	0.012^d^	0.001	0.008^e^	0.005	0.085^f^	0.009	0.120^g^	0.000
*Caffeic acid hexoside*	0.330^a^	0.002	0.354^b^	0.000	0.134^c^	0.000	0.089^d^	0.000	0.132^e^	0.000	0.156^f^	0.000	0.320^g^	0.000
*Cryptochlorogenic acid*	0.192^a^	0.005	0.272^b^	0.020	0.065^c^	0.003	0.044^d^	0.000	0.085^e^	0.004	0.125^a, b, e, f^	0.132	0.210^f^	0.039
*Rutinhexoside*	0.012^a^	0.000	0.015^b^	0.000	0.006^c^	0.000	0.001^d^	0.000	0.002^d^	0.000	0.032^e^	0.000	0.018^f^	0.000
*Cumaroylquinic acid*	0.015^a^	0.001	0.021^b, e^	0.001	0.021^b, d, e^	0.002	0.008^c^	0.001	0.024^d^	0.001	0.020^a, b, c, d, e^	0.011	0.019^e^	0.002
*Feruloylquinic acid*	0.003^a, b^	0.002	0.002^a^	0.000	0.003^a, b^	0.000	0.001^a^	0.000	0.002^a^	0.000	0.001^a^	0.000	0.003^b^	0.000
*Naringenin C hexoside*	0.206^a^	0.004	0.285^b^	0.002	0.074^c^	0.001	0.085^d^	0.001	0.042^e^	0.002	0.065^c^	0.009	0.101^f^	0.007
*Eriodicthiol*	0.058^a^	0.000	0.069^b^	0.001	0.036^c^	0.001	0.049^d^	0.001	0.012^e^	0.000	0.041^c, d^	0.009	0.047^d^	0.005
*Tricaffeoilquinic acid*	0.021^a^	0.003	0.021^a^	0.002	0.012^b^	0.001	0.020^a^	0.003	0.014^c^	0.003	0.014^c^	0.001	0.013^c^	0.001
Caffeic Acid dihexoside ^*^	0.320^a^	0.001	0.360^b^	0.002	0.080^c^	0.002	0.098^d^	0.003	0.150^e^	0.003	0.215^f^	0.003	0.250^g^	0.001
Naringenin C diglycoside ^*^	0.321^a^	0.000	0.496^b^	0.000	0.029^c^	0.001	0.035^d^	0.000	0.052^e^	0.001	0.062^f^	0.001	0.089^g^	0.001
Eriodicthiol O hexoside ^*^	0.089^a^	0.003	0.130^b^	0.003	0.001^c^	0.001	0.009^d^	0.001	0.008^d^	0.001	0.019^e^	0.059	0.028^f^	0.022
Quercetin O dihexoside ^*^	0.021 ^a^	0.001	0.041^b^	0.001	0.001^c^	0.000	0.024^d^	0.001	0.009^e^	0.001	0.009^e^	0.001	0.011^e^	0.001
Rutin O pentoside ^*^	0.725^a^	0.000	0.812^b^	0.002	0.086^c^	0.000	0.081^d^	0.000	0.160^e^	0.001	0.142^f^	0.012	0.236^g^	0.000
Floretindiglycoside ^*^	0.321^a^	0.002	0.486^b^	0.000	0.213^c^	0.000	0.052^d^	0.001	0.280^e^	0.000	0.241^f^	0.001	0.286^g^	0.005
Kaempferol 3-O-rutinoside ^*^	0.063^a^	0.001	0.080^b^	0.001	0.056^c^	0.001	0.012^d^	0.001	0.005^e^	0.000	0.060^a^	0.006	0.075^f^	0.003

*The results are expressed in mg/100 g of fresh weight and reported as mean ± SD from three independent experiments*.

a−g *Different letters show a significant difference (p < 0.05) among the different typologies of canned tomatoes*.

* *Semi-quantification. with rutin*.

### Total Phenolic Content and *in vitro* Bioaccessibility

Total phenolic content was determined using the Folin-Ciocalteu assay, and the bioaccessibility of tomato polyphenols was assessed using an *in vitro* digestion protocol in order to provide valuable insights into their bioaccessibility. Therefore, the TPC content of non-digested samples were compared with that of digested ones. As shown in Table 7, the TPC content in the digested and non-digested samples was quantified to be in the range of 26.317 to 85.638 and 27.895 to 162.597 mg GAE/100 g, respectively. Moreover, the data highlighted that all the digested samples showed significantly lower TPC values (*p* < 0.05) than the digested ones, except for diced tomatoes. The percentage of decrease in TPC following GI digestion ranges from 1.6 (diced tomatoes) to 59.7% (triple concentrate), as indicated in Table 7. Furthermore, the data revealed that five out of the seven samples showed a decrease in the bioaccessibility of polyphenols of <20%, whereas the two remaining samples showed a decrease in polyphenol bioaccessibility between about one-thirds (double concentrate) and two-thirds (triple concentrate) compared with the initial TPC values.

**Table 7:** Total phenolic content of the investigated samples measured in digested and non-digested samples.

**Samples**	**Not digested**		**Digested**		**%**
	**Mean**	**±DS**	**Mean**	**±DS**	
Double concentrate	126.976^a^	2.626	85.638^a^	0.637	67.4
Triple concentrate	162.597^b^	0.783	65.587^b^	0.627	40.3
Diced tomatoes	30.888^c^	0.822	30.383^c^	0.303	98.4
Peeled tomatoes	31.750^c^	0.673	28.487^d^	0.511	89.7
Crushed tomatoes	27.895^d^	0.256	26.317^e^	0.491	94.3
Tomato sauce	37.614^e^	0.139	30.318^c^	0.042	80.6
Cherry tomatoes	41.692^f^	0.28	40.306^f^	0.216	96.7

*Data are displayed as mean of mg GAE/100 g of the samples and standard deviation (SD)*.

a−f *Different letters show a significant difference (p < 0.05) among the different typologies of canned tomatoes*.

### Antioxidant Capacity and *in vitro* Bioaccessibility

The antioxidant capacity of the assayed samples recorded in the initial samples and following GI digestion was evaluated and compared. Three spectrophotometric methods, namely, DPPH, ABTS, and FRAP, were used to monitor variations in antioxidant capacity. The findings are summarized in Table 8. The data highlighted that the digestion process affected the active compounds present in the assayed samples, resulting in decreased antioxidant activity. In fact, compared to the initial values, the samples subjected to simulated GI digestion ended up with a significantly (*p* < 0.05) lower antioxidant activity. In particular, the results of antioxidant capacity revealed lowered values ranging from 36.1 to 89.5% (DPPH test), 13.5 to 53.7% (ABTS test), and 40.4 to 80.5% (FRAP test). Furthermore, the triple concentrate sample was found as the sample that showed highest decrease in antioxidant activity measured in all the three tests performed in this study.

**Table 8:** Antioxidant activity of digested and non-digested canned tomato samples (n = 7).

**Samples**	**DPPH**					**ABTS**					**FRAP**				
	**Not digested**		**Digested**		**%**	**Not digested**		**Digested**		**%**	**Not digested**		**Digested**		**%**
	**Mean**	**±DS**	**Mean**	**±SD**		**Mean**	**±SD**	**Mean**	**±SD**		**Mean**	**±SD**	**Mean**	**±SD**	
Double concentrate	1.308^a^	0.046	0.268^a^	0.023	20.5	2.508^a^	0.076	1.552^a^	0.053	61.9	1.374^a^	0.063	0.387^a^	0.021	27.6
Triple concentrate	1.413^b^	0.035	0.149^b^	0.016	10.5	2.618^a^	0.086	1.212^b^	0.048	46.3	1.676^b^	0.063	0.335^a^	0.033	19.5
Diced tomatoes	0.334^c^	0.027	0.197^c^	0.026	59.0	0.595^b^	0.042	0.453^c^	0.036	76.2	0.338^c^	0.048	0.204^b^	0.031	58.6
Peeled tomatoes	0.339^c^	0.034	0.121^b^	0.015	35.7	0.621^b, c^	0.061	0.538^d^	0.049	86.5	0.383^c^	0.061	0.176^b^	0.027	45.9
Crushed tomatoes	0.319^c^	0.029	0.117^b^	0.017	36.7	0.571^b^	0.057	0.320^e^	0.026	55.9	0.355^c^	0.025	0.214^b^	0.025	55.6
Tomato sauce	0.429^d^	0.016	0.123^b^	0.013	28.7	0.713^c, d^	0.059	0.430^c^	0.036	60.3	0.477^d^	0.039	0.276^c^	0.023	56.5
Cherry tomatoes	0.447^d^	0.023	0.286^a^	0.021	64.0	0.733^d^	0.049	0.522^d^	0.039	71.2	0.545^d^	0.044	0.329^c^	0.030	59.6

*The results are expressed in mmol Trolox/Kg ±DS*.

a−e *Different letters show a significant difference (p < 0.05) among the different typologies of canned tomatoes*.

Furthermore, strong positive correlations between TPC content and antioxidant capacity measured by DPPH, ABTS, and FRAP were observed for the initial values and following the GI digestion process, except for the DPPH test of the digested samples (R^2^ = 0.525), as shown in Table 9.

**Table 9:** Correlation between total phenolic content (TPC) and data obtained by the DPPH, ABTS, and FRAP tests.

**Assay**	**Not digested samples *R*^2^**	**Digested samples *R*^2^**
DPPH	0.964	0.525
ABTS	0.953	0.942
FRAP	0.973	0.858

### Carotenoids Content and Their *in vitro* Bioaccessibility

The carotenoid profile of the most representative compounds such as lutein, β-carotene, and lycopene was quantified using an HPLC method. Calibration curves with real standards at 12 concentration levels were employed (regression coefficient > 0.99) for quantitative determination of the assayed compounds. Table 10 shows the results (mean value and SD) obtained from the initial samples and following simulated GI digestion. In addition, the percentage of bioaccessibility (non-digested vs. digested samples) of each investigated carotenoid was also displayed. In the assayed canned tomato samples here, lycopene was found as the most commonly quantified carotenoid, with concentrations ranging from 50.894 to 222.061 mg/kg. As shown in Table 10, after GI digestion, the amount of lycopene is recorded in the range between 9.3 and 20.0% of the non-digested analyzed samples. As far as β-carotene was concerned, the levels of this important carotenoid were quantified in the assayed canned tomato samples at a concentration range of 9.313 to 52.404 mg/kg. After GI digestion, significant decrease in β-carotene was observed, ranging between 72.4 (crushed tomatoes) to 86.1% (triple concentrate). On the other hand, lutein was the less relevant carotenoid, being quantified in the initial canned tomato samples with a concentration range of 0. 654–4.018 mg/kg. In line with the other assayed carotenoids, decreased levels of lutein were observed after gastrointestinal digestion when compared with values of the non-digested samples.

**Table 10:** Intestinal bioaccessibility of carotenoids evaluated by the HPLC-DAD method in the digested and non-digested canned tomato samples (*n* = 7).

**Samples**	**Lutein**					**β-carotene**					**Lycopene**				
	**Not digested**		**Digested**		**%**	**Not digested**		**Digested**		**%**	**Not digested**		**Digested**		**%**
	**Mean**	**±DS**	**Mean**	**±DS**		**Mean**	**±DS**	**Mean**	**±DS**		**Mean**	**±DS**	**Mean**	**±DS**	
Double concentrate	2.851^a^	0.142	0.301^a^	0.052	10.5	40.622^a^	2.133	7.602^a^	0.423	18.7	222.061^a^	19.123	28.325^a^	1.093	12.8
Triple concentrate	4.018^b^	0.243	0.360^a^	0.068	9.0	52.404^b^	3.138	7.264^a^	0.418	13.9	385.643^b^	23.248	35.740^b^	2.138	9.3
Diced tomatoes	0.892^c^	0.073	0.110^b, c^	0.031	12.3	12.696^c^	0.893	2.522^b^	0.323	19.9	69.395^c^	11.183	13.829^c^	1.286	19.9
Peeled tomatoes	0.654^d^	0.064	0.100^b^	0.011	15.3	9.313^d^	0.544	2.133^b^	0.124	22.9	50.894^d^	9.662	7.421^d^	0.863	14.6
Crushed tomatoes	0.779^c, d, f^	0.071	0.131^c^	0.014	16.8	11.273^c^	0.521	3.110^c, d^	0.215	27.6	60.147^c, d^	9.119	10.005^e^	0.529	16.6
Tomato sauce	1.073^e^	0.084	0.178^d^	0.012	16.6	15.238^d^	0.521	3.519^c^	0.191	23.1	83.272^e^	10.231	12.893^c^	0.391	15.5
Cherry tomatoes	0.830^c, f^	0.062	0.121^b, c^	0.014	14.6	11.859^c^	0.432	2.926^b, d^	0.249	24.7	64.771^c, d^	11.158	12.938^c^	0.628	20.0

*The data are expressed in mg/kg of samples and standard deviation (SD)*.

a−f *Different letters show a significant difference (p < 0.05) among the different typologies of canned tomatoes*.

## Discussion

This study aimed to provide valuable insights into the content of active compounds of different typologies of canned tomatoes. Although many scientific studies have reported several beneficial effects of tomato consumption against various chronic diseases, the bioaccessibility of compounds released during GI digestion has been barely studied to date. The protocol employed to replicate human GI digestion was established recently in the INFOGEST network (29).

### Quantification and Retrospective Analysis of Polyphenol Compounds in Canned Tomatoes by UHPLC-Q-Exactive HRMS

Analysis of hydroxycinnamic acids (chlorogenic, caffeic, caffeic O-hexoside, and ferulic acids), flavonols (kaempferol-3-O-glucoside, rutin, and quercetin), flavanones (naringenin), and phenolic acids (protocatechuic acid) by liquid chromatography coupled to mass spectrometry was performed on the seven typologies of canned tomatoes.

Our results are in line with previous findings. Hydroxycinnamic acid derivatives have been found in tomatoes; in particular, chlorogenic acid is the most commonly reported. Rutin and naringenin have been reported as the main flavonoids found in different varieties of tomato. A recent study conducted by (34) investigated the polyphenol profile of tomatoes crude extract. The results showed that the predominant phenolic acid was represented by chlorogenic acid, with a reported concentration range of 6.77–8.65 mg/kg dry material. Rutin content was reported at a concentration range of between 21.07 and 191.18 mg/kg of dry material. Among other polyphenols, ferulic (0.26–1.96 mg/kg dry material), *p-*coumaric (0.11–0.43 mg/kg dry material), and vanillic acids were also found in the analyzed extracts at a lower concentration.

According to other investigations, (35) reported rutin as the most abundant polyphenol in tomatoes, followed by naringenin, and (36) reported an average range of 119.82 and 36.46 mg/kg fresh weight for rutin and naringenin in tomatoes, respectively. Furthermore, (36) also reported the identification of phenolic acid-O-hexosides, cinnamic acids and derivatives, di- and tricaffeoylquinic acid isomers in tomato. Neochlorogenic and cryptochlorogenic acids, naringenin C-hexoside, apigenin-C-hexoside-pentoside, were also found.

In another study conducted by (37), the polyphenol content of tomatoes was investigated. Chlorogenic acid content fell within the range of 0.79–21.8 mg/kg fresh weight, and naringenin was reported to have a concentration range of 0.5 to 6.9 mg/kg fresh weight.

Moreover, (4) monitored and identified phenolic compounds in tomatoes and different types of processed tomatoes sauce. From this study, it emerges that naringenin is increased in the different types of sauce: from 0.12 to 2.38 mg/100 g dry weight. Rutin content significantly increased in industrial processed sauce when compared to fruit, from 24.8 to 33.8 mg/100 g dry weight (36%). Furthermore, (38) validated a UHPLC–QqQ-MS method for analysis of hydroxybenzoic and hydroxycinnamic acid derivatives, flavonols, and flavanones in various typologies of tomato: fruits, sauce, and juice. Cherry tomatoes had the highest levels of rutin and naringenin. The highest content of naringenin was found in tomatoes sauce, with a concentration level of 206 mg/kg fresh weight. Tomato juice extracts had a lower amount of phenolic compounds than cherry tomatoes and sauce extracts.

During canning or drying process, temperature and processstepscould have an impact on the ultimate content of polyphenols, reducing their concentration in processed meals. Total phenolic and flavonoid content could be released into the surrounding medium. On the other hand, canning could also result in the development of several beneficial substances that are not naturally present in raw foods (39).

### Bioaccessibility of Polyphenol Compounds in Canned Tomatoes by UHPLC-Q-Exactive HRMS

It is widely reported that plant-derived foods are a rich source of phytocompounds with high nutritional value. Certainly, the biological function of the human body depends on the real concentration that reaches the site of absorption. Health benefits of phenolic compounds are dependent on how they are released from the matrix, absorbed in the GI tract, and available for metabolism once consumed (40).

Bioaccessibility, the percentage of compounds liberated from the food matrix during GI digestion and rendered available for absorption in the small intestine, can be estimated using an *in vitro* gastrointestinal digestion technique that includes evaluation of the oral, gastric, and intestinal stages. The bioaccessibility of phenolic compounds is influenced by various factors such as their molecular structure. Polyphenols are subjected to various metabolism reactions such as methylation, glucuronidation, and sulfation after being absorbed (41). Polymeric or glycosylated phenolic compounds must be converted before being absorbed in the small or large intestine. The large intestine represents the site of absorption of some parts of polyphenols. With the exception of flavan-3-ols, which are primarily present in their oligomeric or polymeric forms, most flavonoids are glycosylated in their native form. Flavonoid glycosides are excessively hydrophilic to be absorbed in the small intestine directly by passive diffusion. As a result, they are deglycosylated in the small intestine lumen and passively diffused into enterocytes. The small intestine absorbs dietary phenolic acids, with the rest being changed and absorbed in the colon. Bioactive chemicals are then destroyed by the colon microbiota's esterases, resulting in additional absorbable metabolites (19, 42, 43).

In a study conducted by (44), the bioaccessibility of polyphenolic compounds after digestion was determined. A significant decrease in flavonols (26%) was observed; instead, chlorogenic acid increased (24%). Also, (45) reported an increase (<10%) in total phenolics and flavonoids after the intestinal stage. On the other hand, (46) observed a decrease in all classes of polyphenols during intestinal digestion. (47) reported that after the gastric phase there was a significant decrease in total polyphenol content, and that after the duodenal phase, further increase in total polyphenol content was observed, possibly due to structural transformation of polyphenols.

Even though the amount of phenolic compounds in food is widely recognized, to date, research on the impact of food processing on bioaccessibility is lacking.

### Polyphenolic Content and *in vitro* Bioaccessibility

In order to clarify the polyphenol bioaccessibility of different canned tomato products, in this study, a Folin–Cioclteu assay was performed following *in vitro* GI digestion, and the results were compared with the initial values.

Our results are consistent with those previously reported by (48), which highlighted higher TPC and antioxidant values in concentrated canned tomato products than canned tomato and juices, due to the higher dry matter found in concentrated tomato products. The data revealed that, with the exception of chopped tomato samples, the TPC values recorded following GI digestion were significantly lower (*p* = 0.05) than the initial values in all assayed samples. Our data are consistent with the findings reported by (49), who observed a decrease in polyphenol bioaccessibility ranging between 12 and 96% compared to the initial values in tomato-based products. Moreover, (50) reported an increase of polyphenol bioaccessibility in cherry tomatoes as a result of cooking treatment, suggesting that the thermal process may increase the release of phenolic compounds from the matrix.

On the other hand, the antioxidant activity of the canned tomato samples under investigation was measured in both the digested and non-digested samples. The results showed that the samples subjected to *in vitro* GI digestion had significantly lower antioxidant capacity than the non-digested samples. Similar findings have also been observed by (4), who reported that tomato fruits and industrial and home types of processed sauce showed less antioxidant activity against DPPH, ABTS, and FRAP radical oxidation in the small intestine compared to the initial values. Furthermore, the findings revealed strong correlations between the antioxidant capacity data obtained from the spectrophotometric assays, namely, DPPH, ABTS, and FRAP and the TPC results recorded following simulated GI digestion, highlighting that the performed methods provide valid insights into the active molecules released by the different tomatoes products after the simulated gastrointestinal process.

### Carotenoid Content and *in vitro* Bioaccessibility

A wide range of scientific studies highlighted that consumption of regular tomatoes and tomato products may display protective actions against the incidence of a host of conditions, such as cognitive dysfunction, osteoporosis, cardiovascular disease, and light-induced skin damage (51, 52). The bioaccessibility of tomato carotenoids by *in vitro* GI digestion has been studied for different typologies of canned tomatoes by the HPLC-DAD method. Our findings showed that lycopene, β-carotene, and lutein content in the triple concentrate was significantly higher than that in the other assayed products, which is due to the lower moisture content found in the triple concentrate samples than in the other typologies of canned tomatoes (Table 1). Moreover, the data clearly indicate that the GI process could affect the bioaccessibility of carotenoid. In this study, carotenoid bioaccessibility varied from 9.3 to 20.0, 13.9 to 27., and 9 to 16.8% for lycopene, β-carotene, and lutein, respectively.

Similar outcomes were highlighted by (14) who reported that lycopene bioaccessibility from canned tomatoes was about 21%, whereas higher bioaccessibility was reported in fresh tomatoes (about 28%) and sun-dried tomatoes (about 58%). However, (53) investigated the lycopene content in processed and crude tomatoes and found that both samples had very low lycopene bioaccessibility, with concentration levels ranging from 0.1 to 1.6%. On the other hand, the reported bioaccessibility values of β-carotene and lutein in fresh tomatoes vary in the literature; the percentage of bioaccessibility observed by (54) was 15.5 and 58.6% for β-carotene and lutein, respectively. In contrast, (55) reported *in vitro* bioaccessibility of β-carotene from tomatoes paste, and their findings highlighted that the bioaccessibility of β-carotene was approximately 100%.

## Conclusion

Tomato represents a rich source of dietary nutrients, such as flavonoids, phenolic acids, and represents the major source of lycopene linked with many health benefits, such as anticancer activity and cardiovascular protection effects. The results highlighted the high amount of rutin, naringenin, and chlorogenic acid in the analyzed canned tomato samples. Lycopene content was in the range of 50.894–222.061 mg/kg. Although there is not a daily value for lycopene assumption, based on data from epidemiologic investigations, a regular intake of around 6 mg of lycopene could provide protection. According to these data, consumption of three servings (180 g) of canned tomatoes can contribute approximately to this required intake.

Moreover, the data highlighted that the digestion process affects the active compounds present in the assayed samples, resulting in decreased antioxidant activity, total polyphenol content, and recovery of the analyzed compounds. Until now, only few scientific studies have evaluated the bioaccessibility of phenolic compounds from tomatoes. To our knowledge, this is the first study that investigated the bioaccessibility of polyphenolic compounds in Italian canned tomatoes.

In conclusion, the consumption of tomato and canned derived products could be a valid support to the intake of bioactive compounds. Tomato is an excellent source of nutrients useful in disease prevention and maintaining good health. Since limited information on changes in the bioaccessibility of bioactive compounds during GI digestion of canned tomatoes was reported, our study could be a good support to evaluate which content of bioactive compounds may be really bioaccessible to exercise beneficial effects on human health. Therefore, future *in vivo* studies are needed to confirm the real bioavailability of plasma and tissue concentrations of active compounds, and to confirm the *in vitro* results.

## Data Availability Statement

The raw data supporting the conclusions of this article will be made available by the authors, without undue reservation.

## Author Contributions

LI and AR: conceptualization. SL: methodology and formal analysis. LI, AG, and LC: investigation. AR: resources, project administration, and funding acquisition. LI and LC: writing original draft preparation. MG and AR: writing review and editing and supervision. All the authors have read and agreed to the published version of the manuscript.

## Conflict of Interest

The authors declare that the research was conducted in the absence of any commercial or financial relationships that could be construed as a potential conflict of interest.

## Publisher's Note

All claims expressed in this article are solely those of the authors and do not necessarily represent those of their affiliated organizations, or those of the publisher, the editors and the reviewers. Any product that may be evaluated in this article, or claim that may be made by its manufacturer, is not guaranteed or endorsed by the publisher.
